# GLP-1 Receptor Agonists and Cardiovascular Disease in Patients with Type 2 Diabetes

**DOI:** 10.1155/2018/4020492

**Published:** 2018-04-02

**Authors:** María Isabel del Olmo-Garcia, Juan Francisco Merino-Torres

**Affiliations:** ^1^Mixed Endocrinology, Nutrition and Dietetics Research Unit, University Hospital La Fe, València, Spain; ^2^Instituto de Investigación Sanitaria La Fe, València, Spain; ^3^Spanish Clinical Research Network- (SCReN-) IIS La Fe, PT17/0017/0035, València, Spain

## Abstract

Diabetes mellitus is a chronic disease prevalence of which is high and continually growing. Cardiovascular disease continues to be the leading cause of death in patients with T2DM. The prevention of cardiovascular complications and the cardiovascular safety of treatments should be a primary objective when selecting treatment. Among all the drugs available, the compounds known as glucagon-like peptide-1 receptor agonists (GLP-1 RAs) appear to be not just innocuous in terms of CVD but indeed to be beneficial. GLP-1 RA actions not only translate on an improvement of well-known cardiovascular risk factors such as glycaemic control, dyslipidaemia, weight, or arterial hypertension but also might show benefits on endothelial function, coronary ischaemia, and heart failure. On the other hand, recent clinical trials aimed at studying cardiovascular episodes have been conducted with GLP-1 RAs. Only liraglutide and semaglutide have shown superiority in cardiovascular benefit compared with placebo. Although many of the mechanisms by which liraglutide and semaglutide produce a cardiovascular benefit are still unknown it would be desirable for these benefits to be incorporated into the therapeutic algorithms routinely used in clinical practice. The purpose of this review is to explore GLP-1 RA actions not only in cardiovascular risk factors (glucose, weight, and hypertension) but also the possible effects on established cardiovascular disease.

## 1. Introduction

Diabetes mellitus (DM) is a chronic disease, the worldwide prevalence of which is high and continually growing. It is associated with high morbidity and mortality and is one of the diseases with the greatest impact on public health. Between 1990 and 2010, the number of adults diagnosed with diabetes in the United States tripled, from 6.5 million to 20.7 million, while the total population increased by only 27% (from 178 million to 226 million). The International Diabetes Federation calculated that, in 2015, one in every 11 adults had diabetes (415 million individuals) and estimated that by 2040, the prevalence would be one every 10 (642 million individuals) [1]. The American Diabetes Association (ADA) reports that if the current epidemiological trend continues, by 2050 one in three American adults could have DM [2].

In Spain, the results of the largest epidemiological study ever conducted in the country were published in 2012, Di@bet.es. It revealed that 13.8% of Spaniards (5.3 million individuals) over the age of 18 had type 2 diabetes (T2DM). Of these, almost 3 million had been diagnosed, but 2.3 million—43% of the total—were unaware that they had the disease [3].

DM is not only prevalent; it is a complex chronic disease. It is very closely related to the presence of comorbidities and chronic complications that can be macrovascular, microvascular, or mixed. Macrovascular complications include cerebral and peripheral vascular disease and cardiovascular disease. Microvascular complications include diabetic retinopathy, neuropathy, and nephropathy. Mixed complications are also common such as diabetic foot and erectile dysfunction.

Descriptive studies have noted a gradual decline in complications these recent years. This probably reflects the advances in acute clinical care and improvement in national health services and health education in individuals with diabetes. Nevertheless, cardiovascular disease continues to be the main complication and cause of death in the diabetic patient [4]. Heart failure, with an estimated prevalence of 5%, is also considered a health problem of first order in Spain, despite a lack of proper studies to correctly estimate its impact. It is the main cause of hospitalisation in adults over 65 years and accounts for 3% of hospital admissions and 3.5% of healthcare costs. In 2010, heart failure was responsible for 3% of all deaths in men and in 10% in women [5].

## 2. Cardiovascular Disease and Diabetes

The increase in cardiovascular disease (CVD) in patients with DM was already apparent in the Framingham (1979) [6] and MERIT (1993) clinical trials [7], which suggested that diabetic patients have a two- to fourfold risk of CVD compared to nondiabetic patients. Moreover, CVD in patients with DM is three times more likely to have a fatal outcome compared to the normal population.

Now that the false concept of equivalence of diabetes and CVD has been overcome, it is important to bear in mind the concept of vascular continuum introduced by Dzau et al. in 1991. The concept of vascular continuum describes the inexorable progression of CVD from the presence of risk factors to the development of myocardial infarction, left ventricular hypertrophy, and cardiovascular death. This concept has been changing over the years, especially as a result of acceptance of the effects of the renin-angiotensin-aldosterone system (RAAS), introducing the notion of the cardiorenal continuum. Furthermore, the boundary between macro- and microvascular complications is becoming blurred due to a better understanding of the molecular pathogenic mechanisms of DM [8].

A diabetic patient can be found on very different parts of the CVD spectrum. He may be newly diagnosed or present more advanced disease and have suspected silent CVD or may be progressing towards the terminal stages of a cardiovascular disease. In all these cases with the accompanying constellation of other cardiovascular risk factors (CVRF) (hypertension [HT], smoking, obesity, dyslipidaemia, and so on) [9, 10].

The choice of treatment in a patient with T2DM is complex, not only because of the large therapeutic arsenal currently available but also the multitude of circumstances that must be assessed when selecting the right treatment (efficacy, weight loss, risk status or CVD, side effects, costs, hypoglycaemias, etc.). In addition, CVD and the safety of treatments for T2DM have achieved special prominence in recent years. On 21 May 2007, cardiologist Steve Nissen published a meta-analysis suggesting that, compared to a control group, rosiglitazone treatment showed a statistically significantly higher risk of myocardial infarction and an increase in mortality close to statistical significance. Since rosiglitazone was withdrawn in 2010 due to this potentially harmful cardiovascular effect, studies must now demonstrate cardiovascular safety in all new drugs for the treatment of T2DM [11].

Among all the drugs available, the compounds known as glucagon-like peptide-1 receptor agonists (GLP-1 RAs) appear to be not just innocuous in terms of CVD but indeed to be beneficial.

## 3. Incretins: Glucagon-Like Peptide-1 (Glp-1)

The concept of the incretin hormone system and its relationship with DM dates from the 1970s [12]. The incretins are hormones secreted by cells in the midgut that potentiate glucose-dependent insulin secretion [13].

GLP-1 (GLP-1 7-36) is secreted continuously in both interprandial and prandial periods. Primary biological actions described for intact GLP-1 are mediated by the GLP-1 receptor (GLP-1R). GLP-1 (9-36) metabolite which appears after dipeptidyl-peptidase 4 (DPP-4) action also exhibits its own biological actions [13].

The biological action of native GLP-1 (7-36) and its metabolites GLP-1 (9-36) and GLP-1 (28-36) is under study, as it seems that these metabolites could exhibit their own biological actions independent of those mediated via GLP-1R. The actions exerted by GLP-1 through GLP-1R are the best known and are affected in numerous areas as a result of the wide distribution of GLP-1R in the body [14, 15].

These actions include

1. GLP-1 increases glucose-dependent insulin synthesis and secretion in the pancreatic islets. In animal studies, they show an increase or maintenance of the beta-cell mass. It also decreases glucagon secretion by acting on the alpha cells.
2. GLP-1 acts as a neurotransmitter and can act on both the CNS (satiety and loss appetite) and peripheral nervous system (PNS).
3. GLP-1 delays gastric emptying and inhibits pentagastrin and acid secretion stimulated by food ingestion.
4. GLP-1 has cardiovascular benefits on blood pressure, the vascular endothelium, atherosclerosis progression and inflammation, myocardial ischaemia, heart failure, and so on, which will be discussed in detail below.

## 4. Degradation-Resistant Glp-1 Ras

Multiple GLP-1 RAs have been developed for the treatment of T2DM (Table 1) [10]. New GLP-1 RAs are currently being developed, and some are in very advanced phases (e.g., semaglutide and ITCA 650) (Table 1).

## 5. Beneficial Effects of Glp-1 Analogues on Cardiovascular Risk Factors in Patients with Type 2 Diabetes

### 5.1. Glycaemic Control

Although glycaemic control is associated with reductions in the risk of microvascular complications, the benefits of strict glucose control on macrovascular complications are more questionable. It seems reasonable to think after the VADT, ACCORD, and ADVANCE studies that intensive treatments in patients with established cardiovascular disease failed to show a reduction in cardiovascular episodes. Nevertheless, it should be taken into account that the patients selected in these trials were high cardiovascular risk. In contrast, the UKPDS study showed that patients whose treatment began intensively at diagnosis presented a lower incidence of cardiovascular episodes, even at 10 years, when the HbA1c levels for both groups were similar. This arose the concept of “glycaemic legacy,” which was expanded to the concept of “metabolic legacy” following the STENO 2 trial. It therefore seems clear that the intensive treatment of glycaemia along with other metabolic abnormalities in the early stages of the disease produces a benefit on macrovascular complications that is maintained in the long term [16].

All GLP-1 RAs currently approved for the treatment of T2DM are administered subcutaneously. Depending on their pharmacokinetic properties, they will be administered daily or weekly.

Short-acting GLP-1 RAs (daily exenatide and lixisenatide) are administered in relation to meals. These exhibit large fluctuations in their plasma concentrations, resulting in intermittent activation of GLP-1 RAs, producing a modest effect on both glucose levels between doses and fasting plasma glucose and HbA1c control. Exenatide 10 *μ*g twice daily experiences a drop in HbA1c. Exenatide 10 *μ*g experiences a drop in HbA1c of −0.78% and the 5 *μ*g a drop of −0.4%, both significant against placebo [17, 18]. On the other hand, lixisenatide decreases A1c in about −0.32%. However, they show a higher capacity for delaying gastric emptying and, therefore, greater efficacy in reducing postprandial glucose levels. Lixisenatide showed a better reduction in postprandial blood glucose compared to liraglutide which had a better reduction in fasting blood glucose, which is not surprising considering their half-lives.

Long-acting agonists were developed to prolong their action on the GLP-1R and consequently their pharmacodynamic action. They are administered daily (liraglutide) or weekly (exenatide-LAR, albiglutide, and dulaglutide). In head-to-head studies, they show better efficacy in reducing fasting plasma glucose and HbA1c control compared to short-acting drugs. The DURATION-1 study compared the efficacy of exenatide twice daily with a weekly dose. At the end of the study, both treatment arms showed a clear improvement in HbA1c values: 1.9% for the long-acting compared to −1.5% of the short-acting agonist (*p* = 0.0023). The LEAD-6 study compared treatment with liraglutide (1.8 mg/day with dose escalation) versus exenatide (10 *μ*g/12 hours with dose titration) [19]. After a 26-week follow-up, liraglutide showed a reduction of −1.2%, compared to −0.79%. In the AWARD-1 study, dulaglutide was superior to twice-daily exenatide (−1.51% for dulaglutide 1.5 mg/week, −1.30% for dulaglutide 0.75 mg/week, and −0.99 for twice-daily exenatide) [20]. But, due to tachyphylaxis, they do not exert as much effect on the gastric emptying that affects postprandial glycaemic control.

In summary, with respect to glycaemic control, long-acting agonists are more effective in reducing A1c than short-acting. Within long-acting agonists and glycaemic control, liraglutide 1.8 mg has not been statistically significantly surpassed by any GLP-1 RA in head-to-head comparisons carried out to date.

It is important to note that, in addition to glycaemic control, glycaemic variability is a factor that has sometimes been related with a higher risk of CVD due to increased oxidative stress [21, 22]. Studies with GLP-1 RAs to date have not included this measurement. However, in the 52-week extension trial dual action of liraglutide and insulin degludec in type 2 diabetes (DUAL I), the authors studied the fluctuations in plasma glucose of the combination of insulin degludec and liraglutide (IDegLira) against each of its components separately. A significantly lower number of fluctuations were observed in the interstitial glucose with IDegLira, compared to insulin degludec alone. Furthermore, the liraglutide treatment arm behaved similarly—as regards variability—to the cohort with insulin degludec alone [23]. Glycaemic variability should undoubtedly be a field to explore in trials with GLP-1 analogues, due to its possible impact on cardiovascular morbidity.

### 5.2. Arterial Hypertension

Arterial hypertension (HT) is a very common complication in patients with T2DM. It affects 79.4% of diabetic adults in Spain, according to the Di@bet.es study [24]. Excess weight and obesity, insulin resistance, and hyperglycaemia itself are the main factors associated with its greater presence.

The combination of poor blood pressure (BP) control together with poor glycaemic control considerably increases the risk of developing a myocardial infarction, heart failure, or stroke. According to the UKPDS study, a reduction in systolic blood pressure (SBP) of 10 mmHg results in a 15% reduction in mortality in patients with T2DM. In the ADVANCE study, a reduction of 5.6 mmHg reduced the risk of cardiovascular death by 18%. The HOPE study also showed that a reduction of 2.5 mmHg, with or without a 1 mmHg reduction in diastolic blood pressure (DBP), may reduce the risk of myocardial infarction, stroke, or cardiovascular death by 25% [25].

Clinical trial data so far seems to significantly conclude that treatment with GLP-1 analogues reduces BP values. The mechanism by which this reduction occurs has not yet been clearly identified but may be due to complex regulation. In fact, effects occur early—two weeks after the start of treatment—suggesting that it is a decrease independent of weight loss and that other mechanisms may be involved. One potential mechanism could be direct activation of the GLP-1R in arteries and the renal system, including an improvement in endothelial function, as well as a vasodilator and natriuretic effect by inhibition of the RAAS. However, other mechanisms could be independent of GLP-1R, for example, the activation of nitric oxide by cyclic GMP [25].

None of the trials conducted to date has been specially designed to evaluate the effects of GLP-1 RAs on BP. Nevertheless, several reviews and meta-analyses seem to agree that both exenatide and liraglutide produce a mean decrease of −1 to −5 mmHg compared with placebo and other active comparators [25]. In the DURATION trials, weekly exenatide showed a mean reduction in BP of −3 to −5 mmHg. Moreover, in clinical trials with exenatide, twice-daily dosing also resulted in a significant decrease in SBP compared with placebo (−2.8 mmHg) or insulin (−0.37 mmHg), with larger decreases in those patients who started with SBP > 150 mmHg [26]. In the LEAD studies, liraglutide caused a decrease in SBP of between −2.7 and− 6.6 mmHg [19, 25, 27]. It is important to remember that GLP-1 RAs do not reduce BP in normotensive subjects.

Furthermore, GLP-1 RA treatment is also known to be associated with a slight increase in heart rate, generating a mean increase of +1.86 beats per minute (bpm) compared with placebo and + 1.90 bpm with active comparator. These increases are more evident with liraglutide and extended-release exenatide [28]. The mechanism for this could be related to vagal depression, insulin-mediated activation of the sympathetic system, and the large increase in insulin after the infusion of GLP-1. Although drugs that reduce heart rate have been shown to reduce cardiovascular risk, no harmful effect of this increased rate has been observed with GLP-1 agonists to date.

### 5.3. Dyslipidaemia

Given the insulin resistance and metabolic disorder in patients with T2DM, dyslipidaemia is an important and common comorbidity. The typical lipid profile of a T2DM patient, known as atherogenic dyslipidaemia, includes a decrease in HDL cholesterol (HDL-C) and an increase in LDL cholesterol (LDL-C), total cholesterol, and triglycerides. The combination of dyslipidaemia and poor glycaemic control plays an essential role in the development of atherosclerosis [29]. According to the Quebec Cardiovascular Study, the combination of diabetes, high LDL-C, and high apolipoprotein B confers a 20-fold risk of developing cardiovascular episodes [30].

It is interesting to note that several clinical trials with GLP-1 RAs have described an improved lipid profile due to as yet unknown mechanisms. No clinical trials have been conducted that evaluate the different doses and impact on lipid profiles of each GLP-1 RA. Additionally, most trials were not specifically designed to look at the effect of GLP-1 RAs on lipid profile. The majority are head-to-head trials in which the GLP-1 agonist is compared to placebo or other treatments, such as an active comparator, mainly exenatide and liraglutide.

Exenatide in both twice-daily doses of 5 *μ*g and 10 *μ*g and in the extended-release formulation and liraglutide 1.8 mg have shown a reduction in total cholesterol levels. The lowering effect seems more marked with extended-release exenatide and liraglutide 1.8 mg. In terms of lowering triglyceride values, liraglutide (1.2 mg and 1.8 mg) has been found to be more effective [29].

In a meta-analysis of the LEAD trials (liraglutide clinical development program), it was observed that, in all of them, treatment with liraglutide reduced LDL-C (−7.73 mg/dL), total cholesterol (−5.03 mg/dL), and triglycerides, compared with standard treatment. The LEAD-6 study found a reduction in triglycerides of −15.7 mg/dL, compared with twice-daily exenatide. Moreover, decreases in HDL-C were observed, except in patients on combined treatment with TZD [31].

In the DURATION studies (with extended-release exenatide), reductions of between 4.64 and 34.8 mg/dL were found in total cholesterol compared with standard treatment. These reductions were much greater than with twice-daily exenatide. No changes were observed in HDL-C levels [29].

In a 3-year follow-up trial that compared twice-daily exenatide with placebo, the group treated with exenatide were found to have reductions of −6% in LDL-C values, −5% in total cholesterol, and− 12% in triglycerides [32]. Another study, the EUREXA trial, also showed reductions in triglycerides and improvement in HDL-C with twice-daily exenatide compared to glimepiride [33, 34].

A modest improvement in the lipid profile of a patient with T2DM can produce a significant impact from a clinical point of view; nevertheless, the mechanism has not been clearly identified. One possible explanation could be improved glycaemic control, which would reduce insulin resistance and hepatic triglyceride synthesis. Another possible action could be mediated by GLP-1R in the intestinal mucosa, resulting in reduced secretion of apolipoprotein B48, present in the chylomicrons, with a consequent reduction in plasma triglycerides. The beneficial effects of liraglutide could be related to modulation of the expression of certain genes related to lipid and glucose metabolism [35]. Furthermore, in studies performed with exenatide, this agent was seen to suppress the production of intestinal lipoproteins by acting directly on their synthesis, independently of changes in weight, satiety, or gastric emptying [29]. It is important that new trials should be carried out that include all GLP-1 agonists and their effect on the lipid profile as the primary objective and that they explore the mechanism by which this improvement occurs.

### 5.4. Weight

Obesity contributes to the development of both T2DM and CVD. Modest weight losses of 5%–10% have been found to contribute to changes in glycaemic control, number of medications for controlling CVRFs, the patient's functional activity, and their quality of life. GLP-1 RAs have been shown to improve glycaemic control with an added beneficial effect on weight. Mean weight loss has been estimated at between 0.4 and 5.1 kg. However, this improvement in weight varies between GLP-1 RAs and between individuals, although up to 30% of patients do not lose weight [36].

In the analysis performed in the AMIGO trials, patients on twice-daily exenatide presented a weight loss of between 4 and 4.4 kg after 82 weeks when added to SU [37, 38]; the impact of exenatide on weight was maintained for 3 years (−5.3 kg) [32]. In the DURATION-2 trial, weekly exenatide produced significant weight loss (−2.3 kg) within 26 weeks [39].

In the LEAD trials, liraglutide resulted in a weight loss of between 1 and 3.2 kg in 26–52 weeks when it was used alone or added to metformin or metformin plus rosiglitazone. The weight loss observed with liraglutide was dose-dependent [40–43]. In the SCALE trial, high doses of liraglutide 3 mg resulted in a weight loss of 8.4 kg (8% of weight) at 56 weeks compared with placebo [44].

Lixisenatide (Get Goal Study) showed no significant weight losses compared with placebo when it was used alone or added to metformin or pioglitazone. It did, however, provide a discrete reduction in weight when added to SU (−1.76 kg) [45–47].

Dulaglutide resulted in a weight loss of −1.4 to −3 kg when it was used alone or added to metformin (AWARD-3). The weight loss with dulaglutide was similar to that of metformin when both were used alone and greater than sitagliptin when added to metformin (AWARD-5) [48, 49].

In the HARMONY studies, albiglutide was found to be neutral in terms of weight, both when compared with placebo and when added to metformin, metformin + glimepiride or metformin + pioglitazone (HARMONY 1, 3, and 5) [50–52].

There are few studies comparing the effects of the GLP-1 RAs on weight loss. These data should be compared with caution, due to the heterogeneity of the populations studied and the different treatment combinations used. In summary, liraglutide 1.8 mg results in greater weight loss compared with the other GLP-1 RAs. Systematic reviews and meta-analyses of GLP-1 RAs that have included studies on liraglutide (1.2 and 1.8) and exenatide (daily and weekly) have demonstrated weight loss but have been unable to show a significant difference between the agents and different doses. However, head-to-head trials seem to show that lixisenatide and albiglutide have a weaker effect on weight loss [36].

## 6. Other Possible Benefits in Cardiovascular Risk Factors

### 6.1. Glp-1 and Endothelial Function

Endothelial dysfunction is a pathological process that links diabetic macro- and microvascular disease.

Studies conducted in humans observed that the infusion of native GLP-1 in healthy volunteers improved the blood flow in the forearm induced by the secretion of acetylcholine, as measured by plethysmography. In fasting T2DM subjects with stable coronary artery disease (*n* = 12), a notable improvement was found in endothelial function after the infusion of GLP-1, as demonstrated by an increase in flow-mediated vasodilation of the brachial artery during a hyperinsulinaemic clamp. Similarly, in an observational study of 20 diabetic subjects receiving metformin, exenatide treatment (twice daily) for 16 weeks improved flow-mediated vasodilation of the brachial artery after 5 minutes of ischaemia, as determined by ultrasound, compared with patients receiving glimepiride [13].

It is unclear whether the beneficial endothelial effects that are attributed to native GLP-1 are mediated by an endothelial GLP-1R. Many of these studies do not control for the effects of GLP-1 on increasing insulin secretion and decreasing glucose, so the improvement in endothelial function could be by indirect mechanisms.

The intra-arterial infusion of GLP-1 in obese subjects with metabolic syndrome improved acetylcholine- and sodium nitroprusside-induced forearm blood flow only in the presence of an intra-arterial infusion of insulin. In contrast, infusion of GLP-1 into the femoral artery after fasting in healthy subjects improved the flow, independently of insulin. Moreover, GLP-1 promotes vasodilation of isolated mesenteric arteries in the absence of insulin in a nitric oxide synthase-dependent manner.

Liraglutide attenuates induction of plasminogen activator inhibitor type-1 (PAI-1) and vascular adhesion molecule (VAM) expression in human vascular endothelial cells (hVECs) in vitro. Therefore, it may protect against endothelial dysfunction, an early abnormality in vascular disease in diabetic patients. In vitro studies demonstrated GLP-1R-mediated inhibition of PAI-1 and VAM expression. Liraglutide treatment also increased nitric oxide synthase (eNOS) activity and reduced intercellular adhesion molecule (ICAM-1) expression in the aortic endothelium, another GLP-1R-dependent effect. All these studies therefore identify a potential molecular mechanism of protection by GLP-1R-mediated liraglutide against endothelial dysfunction [53].

Further studies are required to evaluate the direct and indirect actions of the GLP-1 RAs against native GLP-1 on endothelial function or vascular smooth muscle cells in diabetic and nondiabetic subjects. They should specify whether part or all the observed effects attributed to GLP-1 are mediated by GLP-1R, GLP-1 (9-36) or degradation products that exert vasodilatory effects independent of GLP-1R function.

### 6.2. Glp-1 Ras and Coronary Ischaemia

A great many preclinical and clinical studies show that the GLP-1 RAs have a cardioprotective effect. Nevertheless, many of these do not distinguish whether the mechanism by which this effect occurs is direct, through the GLP-1R, indirect via other pathways, or whether they could be potential effects of GLP-1 (9-36).

A beneficial effect of the infusion of GLP-1 (for 72 hours) has been observed in patients with acute myocardial infarction (AMI) and left ventricular dysfunction after reperfusion, with improved ejection fraction and ventricular wall motion. Acute infusion of GLP-1, 30 minutes before a dobutamine stress cardiac ultrasound and 30 minutes afterwards prevented the development of postischaemic myocardial dysfunction [13].

Lønborg et al. investigated the effects of a 6-hour infusion of exenatide compared with placebo during the 15 minutes prior to reperfusion in patients about to undergo a coronary intervention to treat ST-segment elevation myocardial infarction (STEMI). Exenatide was effective in reducing the size of the infarct in relation to the ischaemic area and increased the myocardial salvage index measured by cardiac magnetic resonance 90 days postinfusion. In contrast, patients treated with exenatide had no reduction in mortality or improvement in left ventricular contractility. Post hoc analyses revealed that a trend towards a smaller final infarct size in patients treated with exenatide versus placebo (13 ± 9 versus 17 ± 14 g; *p* = 0.11). This cardioprotection described with exenatide was observed in both diabetic and nondiabetic patients [54]. Complementary evidence of the cardioprotective effect of exenatide was obtained in a study that included 58 patients with STEMI and thrombolysis. Compared to placebo, the exenatide group showed an improvement in left ventricular function at 6 months and a reduction in the infarct size at one month [55].

Liraglutide also demonstrated cardioprotection in a trial with 96 patients with STEMI who underwent percutaneous coronary angioplasty. Liraglutide treatment (0.6 mg for two days, 1.2 mg for two days and 1.8 mg for three days) was compared with placebo, finding a better myocardial salvage index in the liraglutide treatment arm (0.66 ± 0.14 versus 0.55 ± 0.15; *p* = 0.001), smaller infarct size (15 ± 12 versus 21 ± 15; *p* = 0.05), and lower high-sensitivity C-reactive protein levels [56].

Thus, native GLP-1 and GLP-1 RAs together produce favourable effects in patients with coronary artery disease, which were initially attributed to direct effects on the myocardium. New findings that call into question GLP-1R expression in the ventricular cardiomyocytes support the hypothesis that this beneficial effect on the myocardium could be mediated by a process independent of the GLP-1R [13].

### 6.3. Glp-1 Ras and Heart Failure

Promising experimental studies in animal models have generated high expectations of the possible benefit of GLP-1 RAs in patients with T2DM and heart failure. Until the moment, there are no published studies in which the effect on heart failure of GLP 1 agonists is the primary objective. Hospital admissions due to heart failure have been explored as secondary objectives. However, there are several clinical trials (http://www.clinicaltrials.gov) completed and pending for publication which explore this, such as functional impact of GLP-1 for heart failure treatment (FIGHT), liraglutide and heart failure in type 2 diabetes, evaluating the use of exenatide in people with type 2 diabetes and diastolic heart failure, incretin-based drugs and the risk of heart failure, effects of exenatide in type 2 diabetic patients with congestive heart failure, and PROCLAIM: effect of AC2592 administered by continuous subcutaneous infusion in subjects with advanced congestive heart failure.

Meta-analyses of phase II/III clinical trials of exenatide, liraglutide, albiglutide, and dulaglutide have shown that they do not increase the risk of hospitalization for heart failure, confirming the findings of cardiovascular safety trials, which we will review later. The Liraglutide Effect and Action in Diabetes: Evaluation of Cardiovascular Outcome Results (LEADER) trial, in particular, showed a significant 12% reduction in the expanded composite outcome comprising the primary endpoint plus coronary revascularizations and hospitalizations for angina and heart failure. However, there was no significant benefit on heart failure admissions [57]. For the moment, clinical data have demonstrated a neutral effect on the incidence of hospitalization for heart failure; nevertheless, this has to be explored as a primary objective in future trials.

## 7. Current Situation: Cardiovascular Outcome Trials (Cvots) on Glp-1 Receptor Agonists

As mentioned above, as of 2008, specific studies must be conducted in all new drugs for the treatment of T2DM to demonstrate their cardiovascular safety. These are performed in order to prove noninferiority as regards the appearance of MACE (major adverse cardiovascular events) with antidiabetic drugs. A multitude of studies has been performed aimed at demonstrating this noninferiority: TECOS, SAVOR TIMI 53, EXAMINE, ELIXA, EMPA-REG, LEADER, SUSTAIN-6, CANVAS, and EXSCEL (Table 2). Next, we will discuss those that were carried out with GLP-1 receptor analogues: ELIXA, LEADER, SUSTAIN-6, and EXSCEL [58].

### 7.1. ELIXA Study

The ELIXA study (evaluation of lixisenatide in acute coronary syndrome) was the first safety study carried out on GLP-1 RAs and was published in December 2015. A total of 6068 patients were included, randomised to treatment with lixisenatide 10 *μ*g daily (which could be increased to 20 *μ*g at the investigator's discretion) or placebo. The aim of the study was to demonstrate the noninferiority of lixisenatide compared with placebo, both with the standard treatment that they required, on the development of MACE. The primary endpoint was the time to occurrence of any of the following events: death from cardiovascular causes, nonfatal myocardial infarction, nonfatal stroke, or hospitalisation for unstable angina. Other secondary endpoints were a composite of the primary endpoint or hospitalisation for heart failure and a composite of the primary endpoint and hospitalisation for heart failure or coronary revascularisation procedures.

The patients included in this trial were all patients with T2DM who had a myocardial infarction or who had been hospitalized for unstable angina within the previous 180 days (secondary prevention). Mean follow-up was 25 months in each group.

With respect to the primary endpoint results, lixisenatide showed noninferiority to placebo (hazard ratio [HR] = 1.02; 95% confidence interval [CI], 0.89–1.17; *p* < 0.001) but not superiority (*p* = 0.81). Analysing the different components separately, the number of deaths from cardiovascular causes (*p* = 0.85), nonfatal myocardial infarction (*p* = 0.71), nonfatal stroke (*p* = 0.54), and hospitalisation for unstable angina (*p* = 0.81) was also similar in both groups. The same occurred with hospitalization for heart failure (*p* = 0.75), coronary revascularisation, and death from any cause.

Within other CVRFs, a modest but significant between-group difference in the change in body weight from baseline was apparent at 12 weeks (−0.6 kg in the lixisenatide group versus −0.0 kg in the placebo group, *p* < 0.001). This relative weight difference was sustained throughout the follow-up period. A modest relative difference (lixisenatide minus placebo) in systolic blood pressure in the lixisenatide group as compared with the placebo group was sustained throughout follow-up, with an average difference across all visits of −0.8 mmHg (95% CI, −1.3 to −0.3) in favor of lixisenatide (*p* = 0.001).

Thus, lixisenatide showed a neutral cardiovascular profile in patients with type 2 diabetes and a recent acute coronary syndrome [59].

### 7.2. LEADER Study

The LEADER trial, published in July 2016, was conducted to study the cardiovascular effect of liraglutide when added to standard treatment for T2DM. The study included 9340 patients who were randomised to treatment with liraglutide (up to a maximum dose of 1.8 mg/day) or placebo. The primary composite outcome was the time to occurrence of the first MACE: death from cardiovascular causes, nonfatal myocardial infarction, or nonfatal stroke. Secondary outcomes included the time to occurrence of the first event: expanded composite cardiovascular outcome (death from cardiovascular causes, nonfatal myocardial infarction, nonfatal stroke, coronary revascularisation, hospitalisation for unstable angina, or hospitalisation for heart failure), death from any cause, and each of the individual components of the expanded composite cardiovascular outcome.

Mean follow-up was 3.8 years. Individuals included were either patients with high cardiovascular risk (>50 years with established CVD) or >60 years with at least one CVRF. With these criteria, approximately 80% of all the patients included had a history of CVD and was, therefore, on secondary prevention, and 20% was on primary prevention.

With respect to primary outcomes, the liraglutide group had a statistically significant lower risk of MACE compared with placebo (HR = 0.87; 95% CI, 0.78–0.97). With respect to deaths from cardiovascular causes, the risk was also lower in the liraglutide group (HR = 0.78; 95% CI, 0.66–0.93 *p* = 0.007). The risk of death from any cause was also lower in the liraglutide group (HR = 0.85; 95% CI, 0.74–0.97; *p* = 0.02), as was the risk of nonfatal myocardial infarction and nonfatal stroke, but the results were not statistically significant.

Analysis of the secondary composite outcome of microvascular complications (nephropathy and retinopathy) showed that the liraglutide group had lower risk (HR = 0.84; 95% CI, 0.73–0.97; *p* = 0.02). In terms of nephropathy alone, there was also lower risk in the liraglutide group (HR = 0.78; 95% CI, 0.67–0.92; *p* = 0.003), although the risk of retinopathy (HR = 1.15; 95% CI, 0.87–1.52; *p* = 0.33) rose slightly but not significantly, in the liraglutide group (probably related to better early glycaemic control).

Significant differences were also observed as regards other CVRFs between the group treated with liraglutide and the placebo group. Weight loss was −2.3 kg greater in the liraglutide group, together with a greater decrease in SBP (−1.2 mmHg) and DBP (−0.6 mmHg). The liraglutide group showed a mean increase in heart rate of 3 bpm.

In this trial, patients on treatment with liraglutide had a lower risk of presenting the primary outcome and a lower risk of cardiovascular death and death from any cause and microvascular complications, demonstrating superiority in terms of cardiovascular safety. The number of patients needed to treat (NNT) to prevent an episode in 3 years was 66 for the primary outcome and 98 for death from any cause [60].

### 7.3. SUSTAIN-6

The SUSTAIN-6 trial (cardiovascular and other long-term outcomes with semaglutide in subjects with type 2 diabetes) was conducted to determine the cardiovascular safety of semaglutide compared to placebo, both in the presence of standard treatment, and was published in September 2016. Semaglutide is a new GLP-1 RA that has still not been approved for the treatment of T2DM; it has a long half-life (6-7 days), which enables weekly subcutaneous administration.

SUSTAIN-6 was a randomised, double-blind, placebo-controlled trial. It included 3297 patients who were randomised 1 : 1 : 1 : 1 to treatment with semaglutide 0.5 mg, semaglutide 1 mg, or placebo (two doses similar to those of the semaglutide treatment).

The primary composite outcome was the time to occurrence of the first MACE: death from cardiovascular causes, nonfatal myocardial infarction, or nonfatal stroke. Secondary outcomes included the time to occurrence of the first event: expanded composite cardiovascular outcome (death from cardiovascular causes, nonfatal myocardial infarction, nonfatal stroke, coronary revascularisation, hospitalisation for unstable angina, or hospitalisation for heart failure) and death from any cause and each of the individual components of the expanded composite cardiovascular outcome. Retinopathy and follow-up of nephropathy were also assessed.

As in the LEADER trial, patients included in SUSTAIN-6 were patients with very high cardiovascular risks who were ≥50 years old with established CVD (coronary heart disease, cerebrovascular disease, peripheral vascular disease, chronic kidney disease stage III or greater, or heart failure NYHA class II or III) or ≥60 years old with at least one CVRF. Mean follow-up was 2.1 years. Of the 3297 patients, 2735 (83.0%) had established cardiovascular disease (including chronic kidney disease of stage 3 or higher), 1940 patients (58.8%) had established cardiovascular disease without chronic kidney disease, 353 (10.7%) had chronic kidney disease only, and 442 (13.4%) had both cardiovascular disease and kidney disease; 17% of the patients had cardiovascular risk factors and was 60 years of age or older.

With respect to the primary outcome results of the semaglutide group, the first cardiovascular episode presented on 108 occasions (1648 patients; 6.6% of them with at least one episode) compared to 146 episodes in the placebo group (1649 patients; 8%), which implies a HR = 0.74 (95% CI, 0.58–0.95), *p* < 0.001 for noninferiority and *p* = 0.02 for superiority. The first episode of nonfatal myocardial infarction occurred on 47 occasions in the semaglutide group and in 64 in the placebo group: HR = 0.74 (95% CI, 0.51–1.08; *p* = 0.12), a difference that was not significant. With respect to nonfatal stroke, the semaglutide group presented 27 episodes compared with 44 in the placebo group: HR = 0.61 (95% CI, 0.38–0.99; *p* = 0.04). The risk of cardiovascular death was similar in both groups (*p* = 0.92), nor were differences observed in death from any other cause (*p* = 0.79).

Significant differences were also observed as regards other CVRFs between the group treated with semaglutide and the placebo group. The semaglutide group presented a reduction in HbA1c of −1.1% in patients who received the 0.5 mg dose and− 1.4% in those treated with the 1 mg dose, both with significant differences with the placebo group (*p* < 0.001). During the trial, the use of antidiabetic medication in the placebo group was much greater than in the semaglutide group, and they tended to take insulin more than twice as frequently. Weight loss was −3.6 kg greater in the semaglutide 0.5 mg group and − 4.9 kg in the semaglutide 1 mg group. In the placebo group, weight losses of −0.7 kg and− 0.5 kg were observed, respectively. Compared to the placebo group, the weight loss in the semaglutide group was 2.9 kg in those who received doses of 0.5 mg and 4.3 kg in those who received 1 mg (*p* < 0.001). The semaglutide group also presented a decrease in SBP of −1.3 mmHg (0.5 mg) and− 2.6 mmHg (1 mg), compared with placebo (*p* < 0.001). As with liraglutide, the semaglutide group showed an increase in heart rate with respect to placebo of 2 bpm (0.5 mg group) and 2.5 bpm (1 mg) (*p* < 0.001).

Fifty diabetic retinopathy complications occurred in the semaglutide arm and 29 in the placebo arm (HR = 1.76; 95% CI, 1.11–2.78; *p* = 0.02). These differences were observed early in the trial. With respect to retinopathy treatments, photocoagulation was required on 38 occasions in the semaglutide group versus 20 in the placebo group and intravitreal agents on 16 occasions with semaglutide versus 13 with placebo. Complications such as vitreous haemorrhage occurred on 16 occasions (semaglutide) versus 7 (placebo), while 5 (semaglutide) patients versus 1 (placebo) developed diabetes-related blindness. Of the 79 patients with retinopathy complications, 66 (83.5%) had preexisting retinopathy (42 of 50 in the semaglutide group [84%], and 24 of 29 in the placebo group [82.8%]) [61, 62]. Worsening of retinopathy was related to the presence of retinopathy at the start of the study, poor baseline metabolic control and with greater reductions in HbA1c in the first 16 weeks of the trial [63].

As regards the appearance of new nephropathy or worsening of existing nephropathy, there were 62 episodes in the semaglutide arm and 100 in the placebo group (HR = 0.64; 95% CI, 0.46–0.88; *p* = 0.005).

In this trial, patients on semaglutide treatment had a 26% lower risk of developing the primary outcome. This lower risk is attributed above all to the significantly lower risk of developing nonfatal stroke (39%) and a nonsignificant reduction in the risk of developing a nonfatal myocardial infarction (26%), since no differences were observed as regards cardiovascular death. The NNT to avoid this primary event would be 45 for 24 months. Thus, semaglutide shows superiority as regards cardiovascular safety [64].

### 7.4. EXSCEL

The EXSCEL trial (exenatide study of cardiovascular event lowering), published in September 2017, was conducted to demonstrate the cardiovascular safety of extended-release exenatide versus placebo, both administered with standard treatment. This study included the largest number of patients with T2DM among the cardiovascular safety studies conducted with GLP-1 RAs (more than 14,752 patients, in 687 centres in 35 countries) with a wide variety of cardiovascular situations. Patients were randomly assigned in a 1 : 1 ratio to receive subcutaneous injections of extended-release exenatide at a dose of 2 mg or matching placebo once weekly.

The primary outcome was defined as the first occurrence of any component of the composite outcome of death from cardiovascular causes, nonfatal myocardial infarction, or nonfatal stroke (three-component MACE outcome), in a time-to-event analysis. Secondary outcomes included death from any cause, death from cardiovascular causes, and the first occurrence of nonfatal or fatal myocardial infarction, nonfatal or fatal stroke, hospitalization for acute coronary syndrome, and hospitalization for heart failure, in time-to-event analyses.

The trial was designed such that approximately 70% of enrolled patients would have had previous cardiovascular events, and 30% would not have had previous cardiovascular events. Of the 14,752 patients (of whom 10,782), 73.1% had previous cardiovascular disease. The median duration of follow-up was 3.2 years.

Weekly exenatide did not increase the incidence of the first episode of MACE (death from cardiovascular causes, nonfatal myocardial infarction, and nonfatal stroke) compared to placebo (HR = 0.91; 95% CI, 0.83–1.00; *p* < 0.001 for noninferiority). Fewer episodes were observed with exenatide (839; 11.4%) than with placebo (905; 12.2%), but statistical significance was not reached to demonstrate superiority (*p* = 0.061). The rates of the first fatal or nonfatal myocardial infarction, fatal or nonfatal stroke, and other secondary outcomes did not differ significantly between the two groups. Additionally, in a prespecified secondary analysis, patients treated with weekly exenatide had a 14% lower incidence of death from all causes compared to placebo (HR = 0.86; 95% CI, 0.77–0.97). Therefore, the incidence of MACE did not differ between weekly exenatide and placebo [65].

Within other CVRFs, the mean glycated hemoglobin level was 0.7 percentage points lower in the exenatide group than in the placebo group (95% confidence interval [CI], −0.7 to −0.6). Overall, least-square mean values were also lower with exenatide than with placebo with respect to body weight (difference of −1.27 kg), systolic blood pressure (−1.57 mmHg), low-density lipoprotein cholesterol (−1.5 mg per deciliter [−0.04 mmol per liter]), and triglycerides (−1.8 mg per deciliter [−0.02 mmol per liter]); values were higher in the exenatide group than in the placebo group with respect to diastolic blood pressure (difference of 0.25 mmHg).

To conclude, once-weekly administration of extended-release exenatide in patients with type 2 diabetes at a wide range of cardiovascular risk appeared not to cause an increase in their overall cardiovascular risk.

CV safety trials conducted to meet the FDA guidance generally use an efficient trial design that enrolls patients with more advanced atherosclerotic CV risk or established CVD to accrue sufficient events in a timely manner. However, a major limitation of such an approach is that the safety population is not representative of patients in ambulatory diabetes care, thereby raising questions about generalizability. Differences in baseline characteristics of the patient population recruited as well as in trial design and protocol make it difficult to compare results from these trials and inappropriate to reliably assess relative benefits of therapies.

Another notable finding is that the favorable CV outcome benefit observed in LEADER and SUSTAIN-6 contrasts with the null results seen with other GLP-1 RA, lixisenatide, and ELIXA trial, which enrolled patients within 180 days of acute coronary syndrome or EXSCEL trial. Although the exact reasons are not clear, this discrepancy might be related to differences in pharmacokinetic and pharmacodynamic properties. Another explanation for the contrasting results might be the trial differences in the enrollment of lower-risk versus higher-risk patients and between the time of follow-up.

## 8. Conclusions

Recent clinical trials aimed at studying cardiovascular episodes associated with the use of antidiabetics have increased our understanding of the potential effects of drugs for T2DM on cardiovascular risk. Clinical trials conducted with GLP-1 RAs and CVOTs present considerable differences in design and enrolment which limits comparisons among them. Liraglutide and semaglutide showed superiority in cardiovascular benefit compared with placebo, both in the presence of standard treatment. Lixisenatide and extended-release exenatide were neutral, that is, they are safe from a cardiovascular point of view, but for the moment they have not demonstrated to provide any benefit.

Although many of the mechanisms by which liraglutide and semaglutide produce a cardiovascular benefit are still unknown (the antiatherosclerotic action hypothesis is prevailing), it would be desirable for these benefits to be incorporated into the therapeutic algorithms routinely used in clinical practice.

Since cardiovascular disease continues to be the leading cause of death in patients with T2DM, the prevention of cardiovascular complications and the cardiovascular safety of the treatment in individuals who have already developed a cardiovascular episode should be a primary objective when selecting treatment for our patients.

## Figures and Tables

**Table 1:** GLP-1 RAs.

GLP 1 RAs	Brand name	Administration	Action
Exenatide	Byetta®	Twice daily	Short acting
Exenatide-LAR	Bydureon®	Once weekly	Long acting
Lixisenatide	Lyxumia®	Once daily	Short acting
Liraglutide	Victoza®	Once daily	Long acting
Albiglutide	Eperzan®	Once weekly	Long acting
Dulaglutide	Trulicity®	Once weekly	Long acting

**(a):** 

	Drug	Intervention	Primary endpoint\	N	Follow-up time (years)	Mean age	Mean HbA1c levels (%)	Previous cardiovascular status of patients
SAVOR TIMI	Saxagliptin	Add saxagliptin versus placebo to standard of care	CV death, AMI, or stroke	18,206	2.1	≥40	≥6.5	CVD or high cardiovascular risk
EXAMINE	Alogliptin	Add alogliptin versus placebo to standard of care	CV death, AMI, or stroke	5380	1.5	≥18	6.5–11	Acute coronary syndrome within previous 15–90 days
TECOS	Sitagliptin	Sitagliptin versus placebo	CV death, AMI, unstable angina, or stroke	14,724	3	≥50	6.5–11	Preexisting CVD
ELIXA	Lixisenatide	Lixisenatide versus placebo	CV death, AMI, unstable angina, or stroke	6076	2.1	≥30	≥7	Acute coronary syndrome within previous 180 days
EMPA-REG outcome	Empagliflozin	Empagliflozin 10 mg versus 25 mg versus placebo	CV death, AMI, or stroke	7000	3.1	≥18	7–10	Preexisting CVD
LEADER	Liraglutide	Liraglutide versus placebo	CV death, AMI, or stroke	9340	3.8	≥50	≥7	≥50 yrs with preexisting disease: CVD/cerebrovascular/renal disease/HF or ≥60 yrs with high CV risk
SUSTAIN-6	Semaglutide	Semaglutide 0.5 mg versus 1 mg versus placebo	CV death, AMI, or stroke	3299	1.99	≥50	≥7	≥50 yrs with preexisting CVD or ≥60 yrs with high cardiovascular risk
EXSCEL	Exenatide	Exenatide 2 mg weekly versus placebo	CV death, AMI, or stroke	14,752	3.2	≥50	6.5–10	70% preexisting CVD
CANVAS	Canagliflozin	CANVAS Canagliflozin 300 mg versus canagliflozin 100 mg versus placebo	CV death, AMI, or stroke	10,142 (4330 CANVAS, 5812 CANVAS-R)	5.69 (CANVAS) 2 (CANVAS-R)	≥50	7–10.5	≥30 yrs with atherosclerotic disease or ≥50 yrs with 3 or more CV risk factors
		CANVAS-R Canagliflozin 100 mg with optional increase to 300 mg versus placebo						

**(b):** 

Cardiovascular event endpoints	SAVOR TIMI		EXAMINE		TECOS		ELIXA		EMPA-REG		LEADER		SUSTAIN-6		EXSCEL		CANVAS
	Class	HR	Class	HR	Class	HR	Class	HR	Class	HR	Class	HR	Class	HR	Class	HR	Class
Primary composite endpoint MACE	CV death, AMI, or stroke	1 (*p* = 0.99)	CV death, AMI, or stroke	0.96 (*p* = 0.315)	CV death, AMI, unstable angina, or stroke	0.98 (*p* = 0.65)	CV death, AMI, unstable angina, or stroke	1.02 (*p* = 0.81)	CV death, AMI, or stroke	0.86 (*p* = 0.04)	CV death, AMI, or stroke	0.87 (*p* = 0.01)	CV death, AMI, or stroke	0.74 (*p* < 0.001)	CV death, AMI, or stroke	0.91 (*p* = 0.39)	CV death, AMI, or stroke
CV death	Primary endpoint	1.03 (*p* = 0.72)	Primary endpoint	0.85 (*p* = 0.212)	Primary endpoint	1.03 (*p* = 0.71)	Primary endpoint	0.98 (*p* = 0.85)	Primary endpoint	0.62 (*p* < 0.001)	Primary endpoint	0.78 (*p* = 0.007)	Primary endpoint	0.98 (*p* = 0.92)	Secondary endpoint	0.88 (ns)	Secondary endpoint
AMI	Primary endpoint	0.95 (*p* = 0.52)	Primary endpoint	1.08 (*p* = 0.47)	Primary endpoint	0.95 (*p* = 0.49)	Primary endpoint	1.03 (*p* = 0.71)	Primary endpoint	0.87 (*p* = 0.23)	Primary endpoint	0.86 (*p* = 0.046)	Primary endpoint	0.74 (*p* = 0.12)	Secondary endpoint	2.1 (ns)	Secondary endpoint
Stroke	Primary endpoint	1.11 (*p* = 0.38)	Primary endpoint	0.91 (*p* = 0.71)	Primary endpoint	0.97 (*p* = 0.76)	Primary endpoint	1.12 (*p* = 0.54)	Primary endpoint	1.18 (*p* = 0.26)	Primary endpoint	0.86 (*p* = 0.16)	Primary endpoint	0.61 (*p* = 0.04)	Secondary endpoint	0.9 (ns)	Secondary endpoint
Hospitalisation unstable angina	Secondary endpoint	1.19 (*p* = 0.24)	Secondary endpoint	0.90 (*p* = 0.632)	Primary endpoint	0.90 (*p* = 0.42)	Primary endpoint	1.11 (*p* = 0.81)	Secondary endpoint	0.99 (*p* = 0.97)	Expanded primary endpoint	0.98 (*p* = 0.87)	Expanded primary endpoint	0.82 (*p* = 0.49)	Secondary endpoint	2.5 (ns)	
Hospitalization heart failure	Secondary endpoint	1.27 (*p* = 0.007)	Expanded primary endpoint	1.19 (*p* = 0.220)	Secondary endpoint	1 (*p* = 0.98)	Secondary endpoint	0.96 (*p* = 0.75)	Secondary endpoint	0.65 (*p* = 0.002)	Expanded primary endpoint	0.87 (*p* = 0.14)	Expanded primary endpoint	1.11 (*p* = 0.57)	Secondary endpoint	1 (ns)	Secondary endpoint

AMI: acute myocardial infarction; CV: cardiovascular; CVD: cardiovascular disease; HF: heart failure; HR: hazard ratio; MACE: major adverse cardiovascular events.
